# Supplementary material for: High-Density EEG in a Charles Bonnet Syndrome Patient during and without Visual Hallucinations: A Case-Report Study
Source: Cells. 2021 Aug 5;10(8):1991. doi: 10.3390/cells10081991 (PMC8392863; doi:10.3390/cells10081991)
Supplement: Supplementary file 1 [file cells-10-01991-s001.zip › cells-1232824-supplementary.pdf]

Supplementary Material

A. Statistical non-Parametric Mapping, SnPM

Single threshold test for the maximum t-statistic

We here explain the rationale of SnPM: let us assume without loss of generality to have collected an EEG feature (i.e. Lempel-Ziv Complexity), for each electrode in two different conditions (i.e. Resting State and Hallucinations), and that each condition is divided in a group of epochs (30 and 32, respectively). For each electrode, a two-sample t-test between the conditions is conducted and its t-value is collected. As the test is applied to multiple electrodes (183), a single-threshold SnPM procedure is used to assess the significance of each t-test, taking into account the multiple comparison issue. Let us consider the null-hypothesis of no significant *condition*-effect: under the null-hypothesis, the labeling of epochs can be randomly assigned (i.e. a feature estimated during a Resting State epoch can be assigned to an Hallucination epoch and vice-versa). Based on this assumption, 5000 random relabeling are conducted, and the t-values related to each single comparison (i.e electrode) are estimated. For each relabeling, only the maximum t-value (in absolute value, for two-tailed significance assessment), among simultaneous comparisons (i.e. over the electrodes) is kept for further analyses. At the end of the relabeling procedure, the maximum t-value distribution under the null-hypothesis of no significant *condition*-effect is thus obtained. The significance of each original t-value is then calculated as the ratio between the number of t-values of the null-distribution exceeding the original t-value (in absolute value) and the number of relabeling.

B. PSD analysis

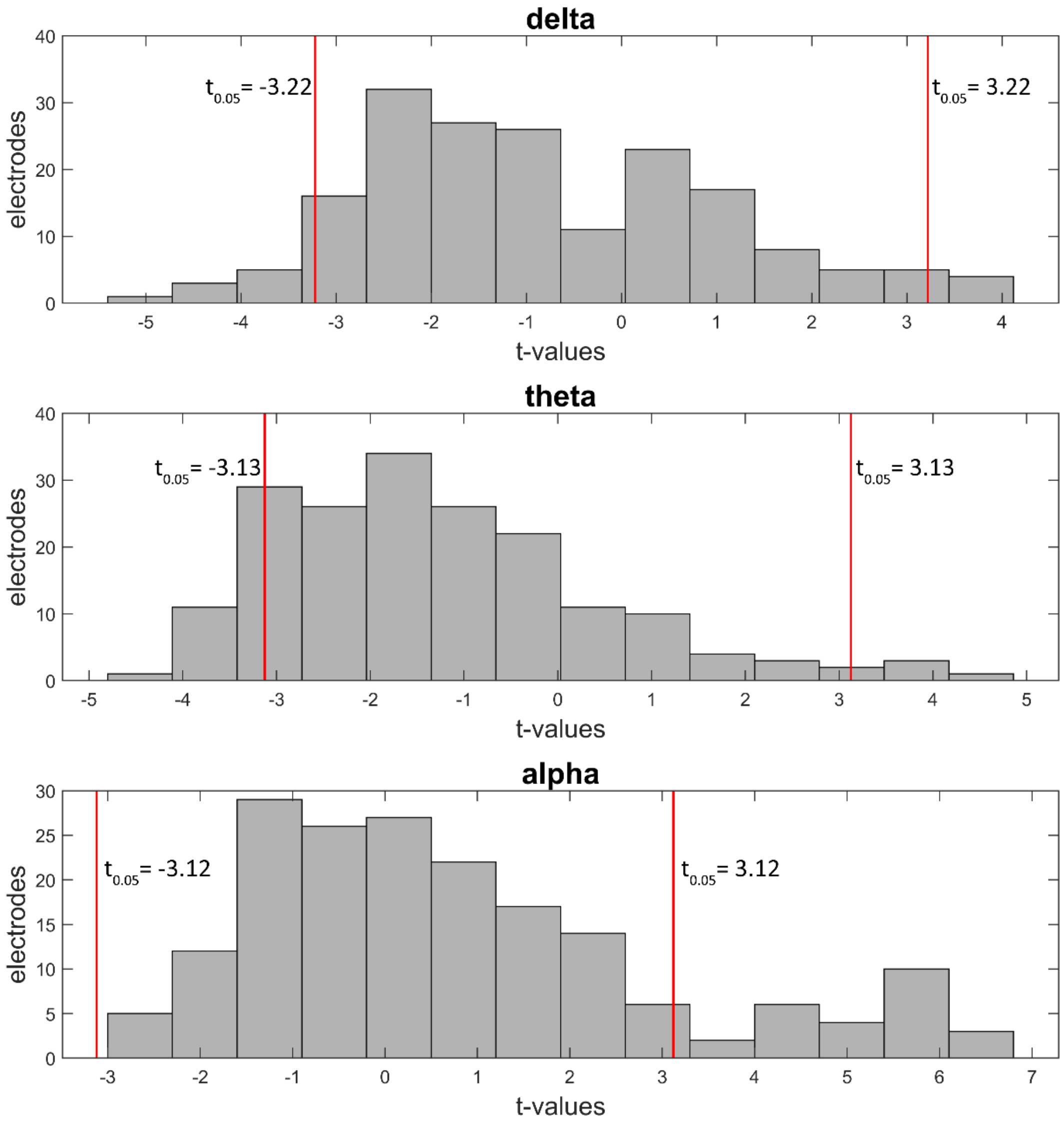

Figure S1. Statistics of the PSD analysis. The distributions of t-values related to the electrode-wise two-samples t-tests are presented for each band (grey bars). In each plot the t-thresholds for significance at $p < 0.05$ (estimated using a single threshold permutation test for the maximum t-statistics, 5000 permutations), are denoted by red lines.

C. Connectivity Analysis

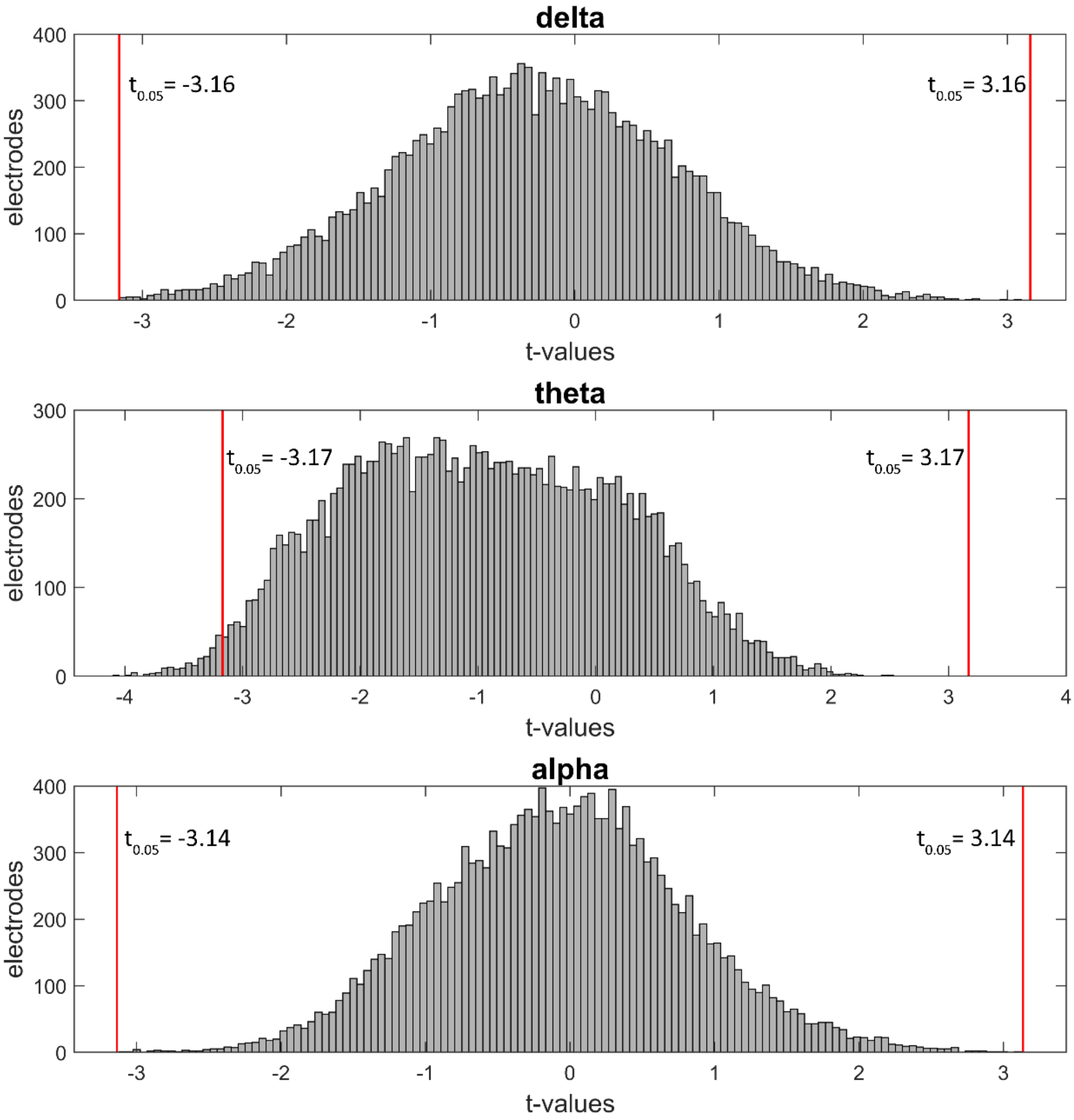

Figure S2. Statistics of the connectivity analysis. The distributions of t-values related to the electrode-couples two-samples t-testsare presented for each band (grey bars). In each plot the t-thresholds for significance at $p < 0.05$ (estimated using a single threshold permutation test for the maximum t-statistics, 5000 permutations), are denoted by red lines.

D. Graph theoretical metrics.

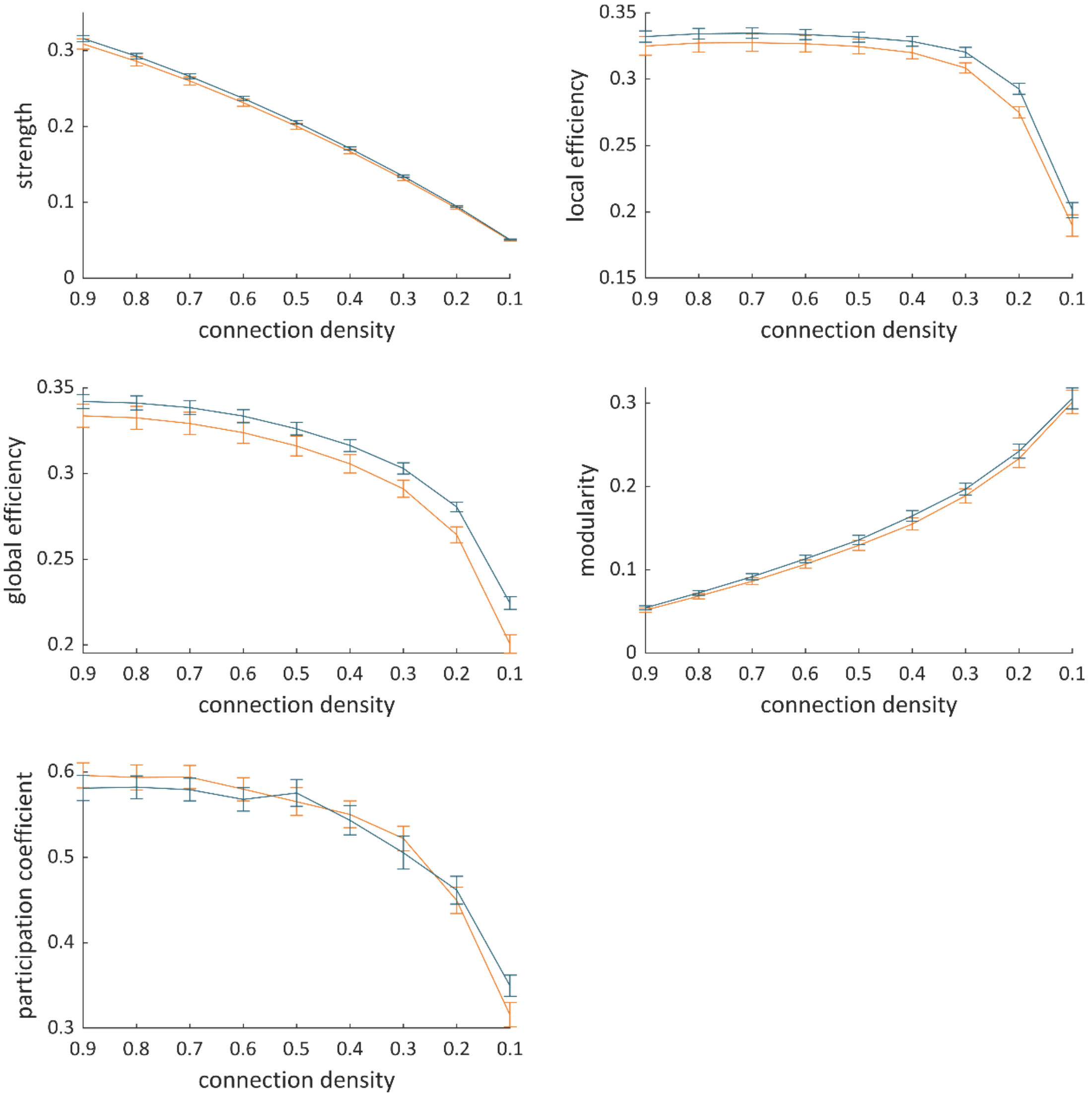

Figure S3. Graph theoretical metrics: Delta. Delta metrics as a function of connection density. Orange lines identify hallucinations condition while blue lines resting state condition. Error-bars denote the mean ± standard error intervals.

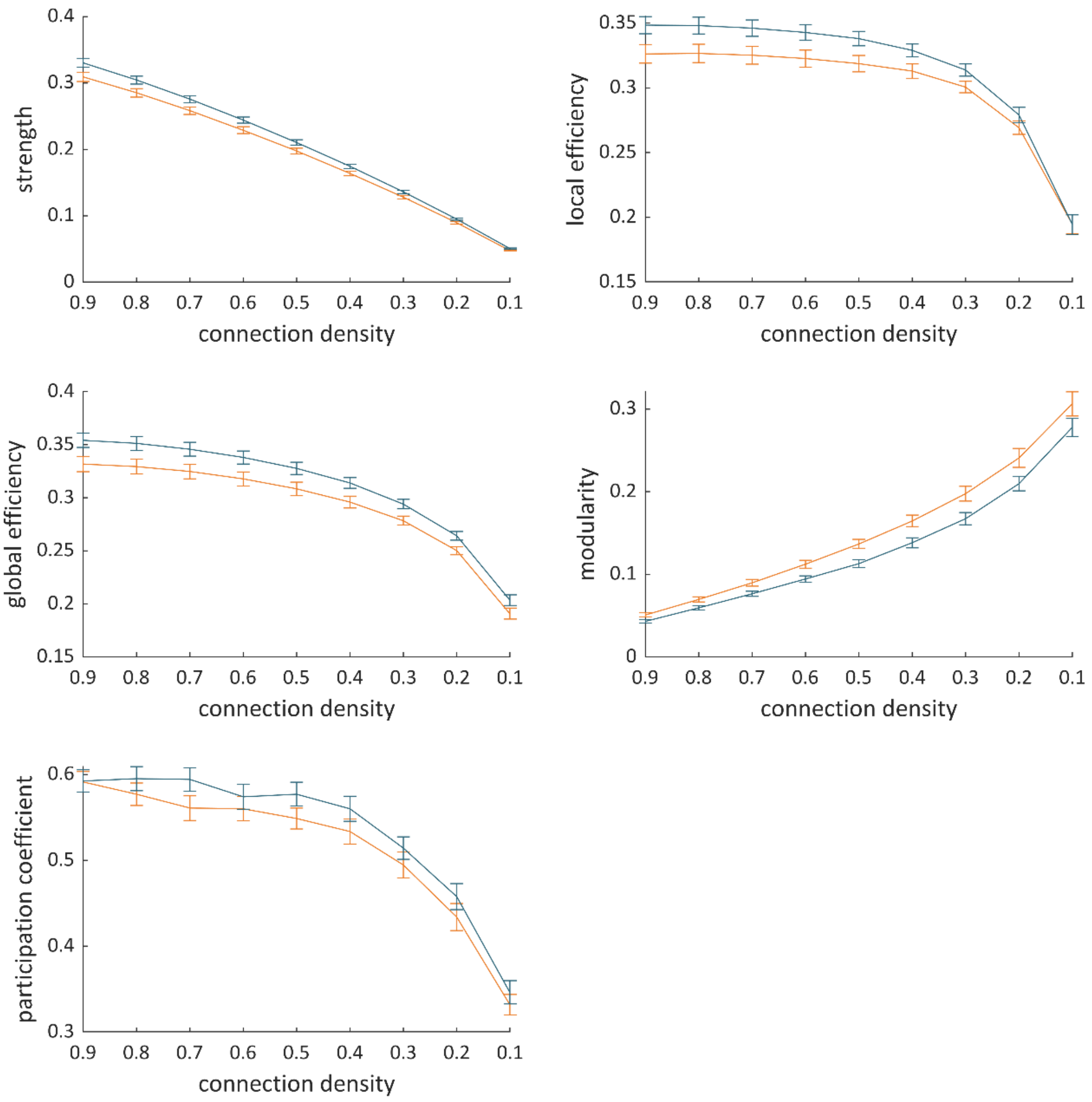

Figure S4. Graph theoretical metrics: Theta. Theta metrics as a function of connection density. Orange lines identify hallucinations condition while blue lines resting state condition. Error-bars denote the mean ± standard error intervals.

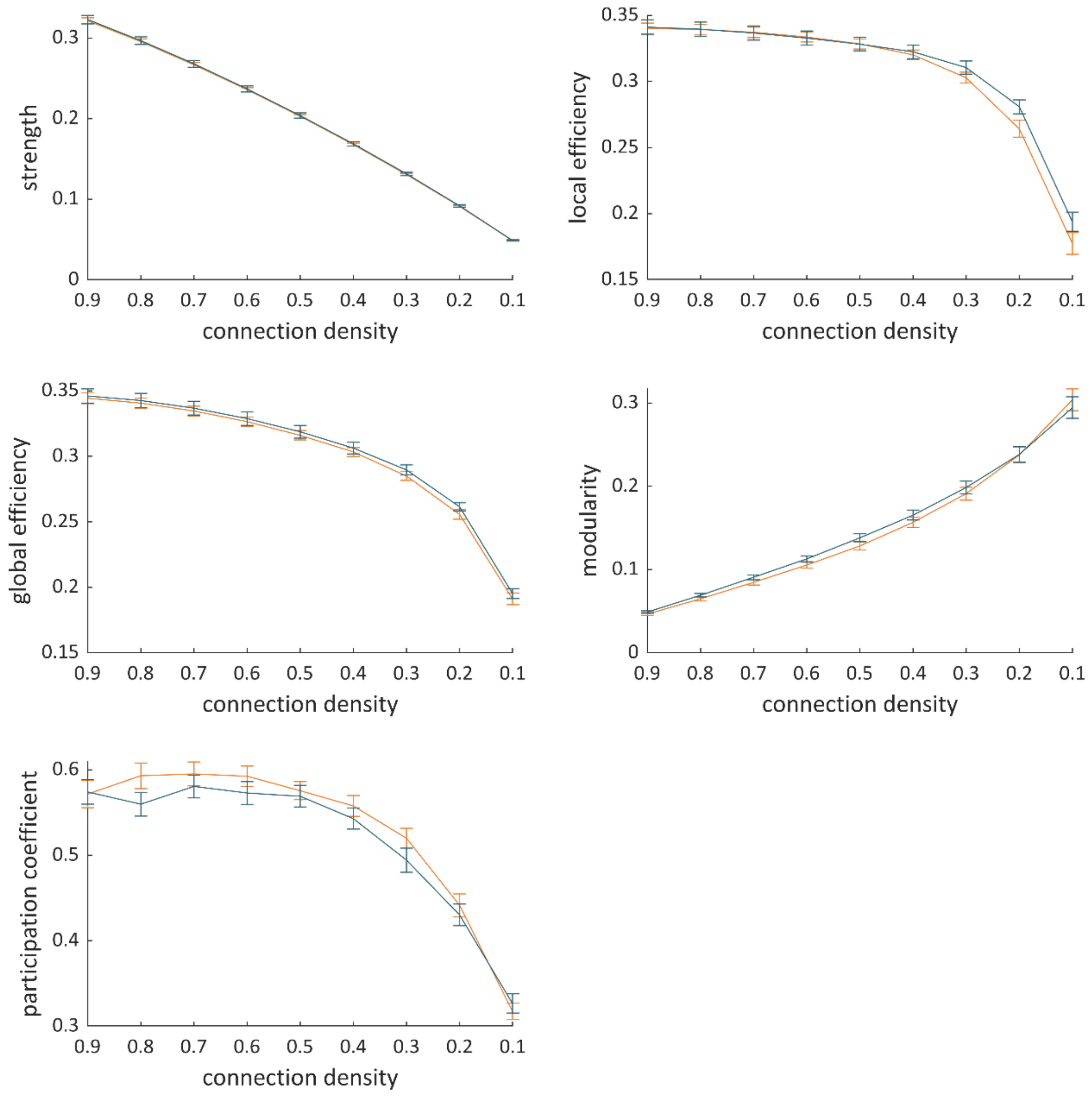

Figure S5. Graph theoretical metrics: Alpha. Alpha metrics as a function of connection density. Orange lines identify hallucinations condition while blue lines resting state condition. Error-bars denote the mean ± standard error intervals.

**DELTA**	**$\|t_{0.05}\|$**	**t-value**	**p-value**	**p_{FDR}**
strength	2.00	-1.03	0.32	0.54
local efficiency	2.05	-1.80	0.09	0.23
global efficiency	2.00	-1.73	0.09	0.23
modularity	2.01	-0.78	0.45	0.57
participation coefficient	1.99	0.27	0.79	0.79

Table S1. Delta networks. Statistics of between-condition comparisons (visual hallucinations versus resting-state) are reported for graph each metric. $|t_{0.05}|$ indicates the two-sided significance threshold (at $p < 0.05$) derived by a permutation test on the t-statistics (5000 permutations), t-value, the t-statistics of the two sample t-test, p-value the non-corrected significance of the test (based on the permutation test), and p_{FDR} the significance after applying Benjamini-Hochberg procedure

**THETA**	**$\|t_{0.05}\|$**	**t-value**	**p-value**	**p_{FDR}**
strength	*2.00*	*-2.22*	*0.03*	*0.04*
local efficiency	*1.99*	*-2.32*	*0.02*	*0.03*
global efficiency	*1.97*	*-2.44*	*0.02*	*0.03*
modularity	*2.02*	*2.72*	*0.01*	*0.03*
participation coefficient	2.03	-1.39	0.18	0.18

Table S2. Theta networks. Statistics of between-condition comparisons (visual hallucinations versus resting-state) are reported for graph each metric. $|t_{0.05}|$ indicates the two-sided significance threshold (at $p < 0.05$) derived by a permutation test on the t-statistics (5000 permutations), t-value, the t-statistics of the two sample t-test, p-value the non-corrected significance of the test (based on the permutation test), and p_{FDR} the significance after applying Benjamini-Hochberg procedure. Metrics showing significant between-condition differences are written in italics.

ALPHA	$|t_{0.05}|$	t-value	p-value	p_{FDR}
strength	2.00	-0.26	0.80	0.80
local efficiency	1.97	-0.79	0.44	0.74
global efficiency	2.00	-0.56	0.59	0.74
modularity	1.98	-0.72	0.49	0.74
participation coefficient	1.95	1.00	0.31	0.74

Table S3. Alpha networks. Statistics of between-condition comparisons (visual hallucinations versus resting-state) are reported for graph each metric. $|t_{0.05}|$ indicates the two-sided significance threshold (at $p < 0.05$) derived by a permutation test on the t-statistics (5000 permutations), t-value, the t-statistics of the two sample t-test, p-value the non-corrected significance of the test (based on the permutation test), and p_{FDR} the significance after applying Benjamini-Hochberg procedure. Metrics showing significant between-condition differences are written in italics.

E. Lempel-Ziv Complexity

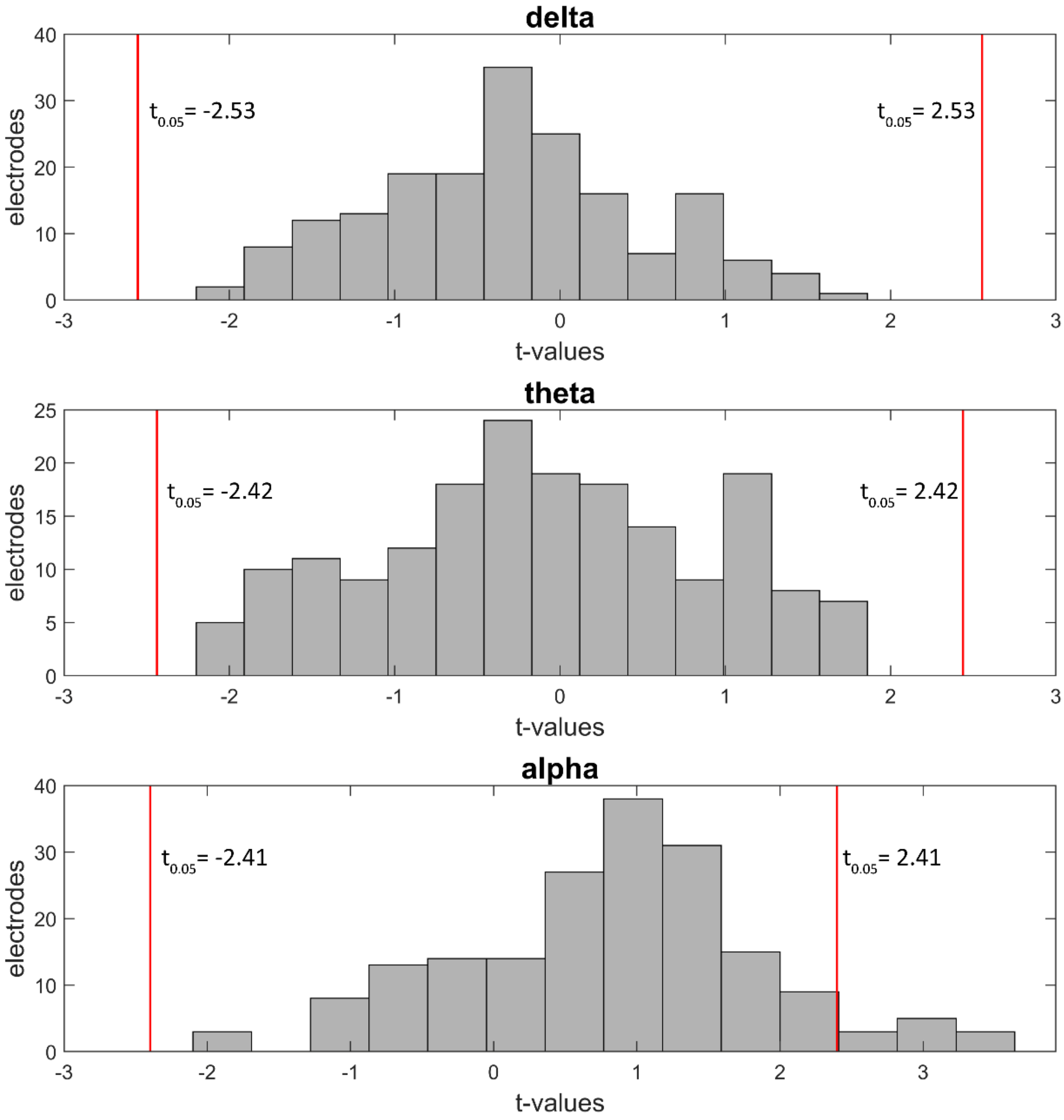

Figure S6. Statistics of the Lempel-Ziv Complexity. The distributions of t-values related to the electrode-wise t-test are presented for each band (grey bars). In each plot the t-thresholds for significance at $p < 0.05$ (estimated using a single threshold permutation test for the maximum t-statistics, 5000 permutations), are denoted by red lines.
